# Effective and Acceptable Eco-Driving Guidance for Human-Driving Vehicles: A Review

**DOI:** 10.3390/ijerph19127310

**Published:** 2022-06-14

**Authors:** Ran Tu, Junshi Xu, Tiezhu Li, Haibo Chen

**Affiliations:** 1 School of Transportation, Southeast University, Nanjing 211189, China; litiezhu@seu.edu.cn; 2 Department of Civil and Mineral Engineering, University of Toronto, Toronto, ON M5S 1A1, Canada; junshi.xu@mail.utoronto.ca; 3 Institute for Transport Studies (ITS), University of Leeds, Leeds LS2 9JT, UK; h.chen@its.leeds.ac.uk

**Keywords:** eco-driving, human-driving vehicles, user acceptance, literature review

## Abstract

Eco-driving guidance refers to courses, warnings, or suggestions provided to human drivers to improve driving behaviour to enable less energy use and emissions. This paper reviews existing eco-driving guidance studies and identifies challenges to tackle in the future. We summarize two categories of current guidance systems, static and dynamic, distinguished by whether real-world driving records are used to generate behaviour guidance or not. We find that influencing factors, such as the content of suggestions, the display methods, and drivers’ socio-demographic characteristics, have varied effects on the guidance results across studies. Drivers are reported to have basic eco-driving knowledge, while the question of how to motivate the acceptance and practice of such behaviour, especially in the long term, is overlooked. Adaptive driving suggestions based on drivers’ individual habits can improve the effectiveness and acceptance while this field is under investigation. In-vehicle assistance presents potential safety issues, and visualized in-vehicle assistance is reported to be most distractive. Given existing studies focusing on the operational level, a common agreement on the guidance design and associated influencing factors has yet to be reached. Research on the systematic and tactical design of eco-driving guidance and in-vehicle interaction is advised.

## 1. Introduction

On-road transportation is an important source of greenhouse gas (GHG) and air pollutant emissions among all economic sectors [1,2,3]. Numerous studies have investigated the factors that influence the amount of vehicle emissions and fuel consumption and proposed control strategies accordingly. From the perspective of vehicles, technologies such as emission after-treatment and vehicle fleet electrification can lead to a substantial decrease in emissions and fuel consumption [4,5,6,7,8]. Methods that mitigate traffic congestion, such as adaptive signal timing control, countdown signal timers, predictive cruise control, and connected automated vehicles (CAVs) can also effectively control traffic emissions through shorter delays and less stop-and-go operations [9,10,11,12]. Compared to the above-mentioned methods, adopting ecological driving behaviour (eco-driving) is a more cost-effective method because it does not require the upgrade of the vehicle powertrain or the reconstruction of infrastructure. Instead, vehicle operational emissions and fuel consumption can be decreased by correcting the driving trajectory.

Many eco-driving studies of road transport focus on the trajectory control of vehicles through connected and autonomous vehicle (CAV) technologies. Scenario-specific studies were proposed to optimize speed and acceleration profiles when vehicles were approaching signalized intersections or driving on highway links. Look-ahead traffic conditions, signal control information, and road geometries are delivered to CAVs through V2X (Vehicle to X communication), and vehicle speed can be dynamically adjusted with the objective of optimal fuel consumption [13,14,15]. However, the speed–time profile determined by eco-driving algorithms cannot be practiced precisely when vehicles are operated by human drivers. Therefore, it is crucial to effectively guide drivers to modify their aggressive driving behaviour, thus approaching the optimal driving operation. The definition of eco-driving behaviour is varied among previous studies. In general, it can be summarized as the following “Golden Rules” [16,17,18], including keeping a steady speed, anticipating surrounding traffic flow, braking smoothly, reducing long-time idling, and avoiding the overuse of auxiliary devices. In the real world, however, due to diverse drivers’ habits and varied traffic conditions, more practical eco-driving guidance should be provided on operating vehicles properly with the purpose of saving energy and reducing emissions instead of strategic guidelines.

This paper aims to present an overview of existing studies on eco-driving guidance for human-driving vehicles of road transport, focusing on the effectiveness and acceptance of the guidance. Different presence formats, their effects on drivers’ behaviour, and associated influencing factors are discussed. Based on the overview, this paper concludes with challenges and research gaps in existing studies, highlighting efforts that can be made in the future to develop an effective and acceptable eco-driving guidance system.

## 2. Designs of Eco-Driving Guidance

**Figure 1:**
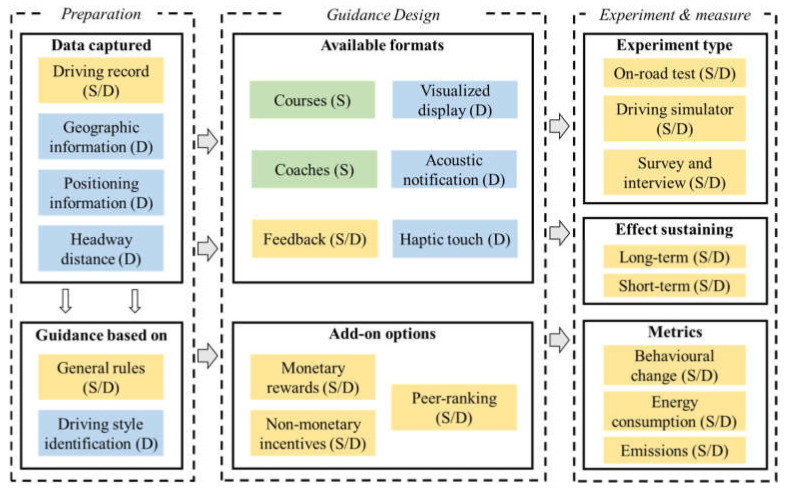
System elements and experiment design of two types of eco-driving guidance (where “S” means the item is only available in static guidance, “D” means the item is only available in dynamic guidance, and “S/D” represents the item is applicable for both static and dynamic guidance).

Existing research presents two types of eco-driving guidance: static training and dynamic guidance. The major difference between them is the use of real-time driving evaluation. The design of systems and the experiments conducted in existing studies are presented in Figure 1. For static training, driving suggestions are provided to drivers through training courses, videos, or education brochures. These suggestions are based on the widely recognized definition of eco-driving, such as the “Golden Rules”. Drivers are first trained by self-learning or coaches; then, they apply the eco-driving knowledge to their daily driving activities. For dynamic guidance, however, driving suggestions are generated based on real-time driving behaviour analysis. Monitoring devices, such as Global Positioning Systems (GPSs) and On-Board Diagnostics (OBD), are equipped in-vehicle. Instantaneous speed and acceleration trajectories are collected. Some experiments also record driving operations such as braking pedal, accelerator pedal, and gear shifting. In this case, energy consumption is usually recorded due to the accessibility of the CAN-BUS data. The analysis of energy consumption or emissions during the recorded time period is then conducted, and corresponding driving suggestions (or warnings) can be generated. The term “dynamic” means that driving suggestions are subject to change with the periodic evaluation of emission reductions or energy savings. In this section, two types of eco-driving guidance strategies and representative examples are discussed in terms of aspects of their effectiveness and drivers’ acceptance with a focus on dynamic guidance.

### 2.1. Static Eco-Driving Training Based on Pre-Determined Guidelines

Static eco-driving training provides driving suggestions based on widely recognized eco-driving guidelines (such as the “Golden Rules”) to drivers through training courses, videos, brochures, and in-field practice with coaches. The training effect is evaluated by monitoring drivers’ behaviour and comparing fuel consumption or emissions before/after the training session. Eco-driving training programs have been implemented within different jurisdictions worldwide [16,19,20]. The change in driving behaviour is measured by comparing fuel consumption and emissions or by comparing key indicators of eco-driving, such as the average speed, acceleration or deceleration rate, frequency of idling, and engine speed (rotation per minute, RPM). Eco-driving training programs are affected by a large range of factors, such as participating vehicle types, road geometry, drivers’ socio-demographic characteristics, and the tested time span; therefore, their effects on behavioural change, energy savings, and emission reductions greatly vary among programs. Table 1 lists existing literature testing the effect of static eco-driving guidance.

### 2.2. Dynamic Guidance Based on Real-Time Driving Operations

Static eco-driving training sessions deliver pre-determined eco-driving rules to drivers and require drivers to apply them during real practice, while the effectiveness of the learning process is questionable, and behavioural change toward eco-driving is hard to maintain for a long time [34,35]. Instead, dynamic eco-driving guidance evaluates real-time driving performance based on driving operations, energy efficiency, and emissions. It generates personalized driving suggestions to drivers via in-vehicle or mobile driving assistance. The in-vehicle assistance device comprises data sensors (such as OBD and GPS) and a module delivering the feedback to drivers. Dynamic eco-driving guidance can be developed using two methods. First, the system records and evaluates on-road driving behaviour and sends a periodic personal driving report through in-vehicle devices or users’ mobile devices. Second, the system generates instantaneous information, such as warnings or speed suggestions, and presents it to drivers through visualized, acoustic, or haptic notifications. In this way, real-time and specific feedback can be provided to drivers according to their driving habits and on-road performance. Existing studies proposed different forms of the information module, varied by the type of feedback and the method to deliver the information. Consequent energy savings and emission reductions depend on the acceptance and practice of drivers, which are related to not only the information type but also drivers’ personality and psychological factors. The results of the use of dynamic eco-driving guidance in existing studies are presented in Table 2.

#### 2.2.1. Periodic Reports and Feedback

A periodic driving report usually includes the evaluation of driving behaviour and suggestions for eco-driving improvement based on real driving records, and the report is regularly pushed to the in-vehicle devices or users’ mobile devices. It can be regarded as a special type of eco-driving training, as a periodic report is more dynamic and customized to drivers’ individual habits. The frequency of the feedback can be daily [37], weekly [45], monthly [37,47,60], or varied by drivers [49], and it is an essential factor that affects the learning process of drivers toward eco-driving skills. For example, Ando and Nishihori [37] revealed that compared to daily feedback, sporadic feedback pushed monthly or weekly may have better performance in encouraging drivers to adopt eco-driving behaviour. In contrast, Wu et al. [48] presented a negative linear relationship between fuel consumption and the frequency of drivers checking the driving feedback.

#### 2.2.2. Combined Sensory Methods and Their Acceptance

Studies and applications have integrated the eco-driving guidance into the vehicle’s human–machine interface (HMI), and the information is delivered through various sensory methods. Visualized suggestions include practical driving recommendations (such as the optimal speed and acceleration), warnings of improper behaviour (such as speeding, excessive braking, or long-time idling), indicators showing the gap between current behaviour and the optimal one, and indicators of environmental performances (such as energy efficiency or emissions). Existing research has also tested auditory notifications and haptic touch in dynamic eco-driving guidance with expectations of enhanced guidance effects [39,41,61].

Haptic touch (including haptic stiffness and haptic force) is the most effective and easy-to-do method. Through pressure on the acceleration pedal or the braking pedal, drivers can easily sense the guidance and follow the instructions [51,52,62,63]. With multiple types of presences being examined (such as dashboard message and colour scale), the visualized system has been reported to be impractical and distractive with additional workload [38,43,55,64]. The auditory system is less disregarded than its visualized alternatives [65], while its user experience is less satisfying [51]. Although eco-driving guidance through haptic touch causes less distraction [66,67], safety concern is still one of its disadvantages [63,66]. The influence of these sensory methods on users’ acceptance, potential safety issues, and environmental benefits need to be further discussed.

#### 2.2.3. Gamification Design in Dynamic Eco-Driving Guidance

Due to the strength in experiential learning environments and the higher satisfaction levels of users [58,68], the concept of “gamification” has gradually attained attention in the eco-driving guidance design. The gamification design provides intrinsic motivation and extrinsic motivation to users to improve their behaviour [69], and the elements in existing applications are points (scores), progress feedback, and socialization [70]. Significant reductions in CO_2_ emissions and energy consumption can be observed in drivers who utilize gamified guidance or additional incentives [53,57,58,71,72].

However, the effect of gamification is debatable. Fitz-Walter et al. [73] observed that a gamified design improved drivers’ satisfaction with the guiding system, while significant behavioural changes did not occur. Some studies also oppose the gamified design as they have shown insignificant results from financial incentives (for example, [26,74]). Furthermore, some researchers have expressed their worries about the negative impact of gamification: the behavioural change encouraged by monetary incentives cannot be sustained upon removal [75], and peer competition through a ranking scheme may lead to an overly competitive situation and cheating in practice [36]. In the current stage, gamification designs used in driving guidance are mainly limited to traditional PBLs (Points, Badges, and Leaderboards), while other elements and drivers’ acceptance of these elements have seldom been investigated [76]. A systematic design of gamified eco-driving guidance should be further explored, and the long-term effect of various gamified elements should be compared.

#### 2.2.4. Optimized Driving Suggestions Considering Traffic States

To improve the anticipation of traffic and drivers’ acceptance of driving guidance, researchers integrated in-vehicle telecommunication systems to retrieve real-time traffic conditions, optimize instantaneous speed and acceleration, and generate eco-driving suggestions dynamically. Downstream signal state, location information, and the motion of surrounding vehicles can be captured from a digital map and a front camera, and optimized guidance shows a great fuel-saving potential (up to 41.9%) [17,40,42,56,59,77]. In addition, by learning the historical driving trajectory of a driver, the shortest learning path can be optimized for the driver, which reduces the acceptance burden when practicing eco-driving operations [54]. These studies demonstrate the environmental benefits of such optimized eco-driving guidance, while large-scale on-road tests are lacking in current studies.

### 2.3. Factors That Affect the Guidance Effectiveness

#### 2.3.1. Vehicle Types and Road Types

Due to different vehicle powertrain and operation requirements, driving habits and associated behavioural changes vary among drivers of different vehicle types after static eco-driving training. The difference in CO_2_ emissions and fuel efficiency is more significant among gasoline vehicles than diesel vehicles and hybrid vehicles [21,29]. In addition, vehicles with manual transmission perform better in terms of saving fuel than automated-transmission vehicles [28].

The driving behaviour on city road segments and major arterials features frequent stop-and-go operations, which greatly influences fuel efficiency and emissions. In other words, the potential for eco-driving improvement on such roads can be more significant than that in highway links [78]. Existing studies also prove that the energy savings and emission reductions after eco-driving training sessions are more significant on congested road links, such as arterial and local streets, while on highway links, where the speed is high and steady, the effect is less significant [22,28,29].

#### 2.3.2. Drivers’ Characteristics

Drivers’ socio-demographic characteristics lead to heterogeneous effects on fuel consumption and emissions among the population, while current studies have not reached a common agreement on how these factors are associated with the effectiveness of eco-driving training. More specifically, Ho et al. [19] found that male drivers have more notable behavioural changes with lower average speeds after the eco-driving training program in Singapore, leading to higher fuel efficiency (15.98% versus 11.21% for female drivers). Unlike the findings of Ho et al., female drivers in the study by Barla et al. [28], Quebec, Canada, had a higher probability of applying eco-driving techniques. Abuzo and Muromachi [23] found that drivers from different jurisdictions performed differently after the same eco-driving guidance, revealing the potential impact of cultural background.

The eco-driving training program is easier to implement because of regular tests and training sessions organized by companies or local authorities. It is worth investigating the effect of eco-driving training in heavy-duty vehicles due to a remarkable share of emissions from these vehicles [79]. Eco-driving guidance programs have been implemented among bus and truck drivers, showing significant fuel drops [24,25,32]. However, several studies have illustrated the difficulty of modifying the driving behaviour and habits of experienced employed drivers due to insufficient monetary reward, less motivation, and strict service time restrictions. For example, Díaz-Ramirez et al. [27] found that more experienced drivers are not likely to change their behaviour after receiving eco-driving guidance, while other socio-demographic characteristics (such as age and education levels) are not significant. Similar results can also be found in [26,33,80].

#### 2.3.3. Sustaining Eco-Driving Behaviour after the Guidance

Since eco-driving is not an automatized, natural, or “everyday” driving style [81], drivers maintaining eco-driving behaviour instead of returning to their “old habits” is essential for the training to have a sustainable environmental benefit. Several studies compared short-term behavioural changes with long-term driving habits [28,35,82], demonstrating a fading effect of the training along the time span. For example, Barla et al. [28] assessed the fuel-saving effect of eco-driving training immediately after the training session and ten months after the session. The reduction in fuel consumption on arterials became 2.5% after ten months, compared to 4.6% immediately after the session. In addition, interruptions during driving can also lead to inconsistent eco-driving behaviour, stressing the difficulties of maintaining the training effect [81]. These studies pose challenges in encouraging drivers to continuously adopt eco-driving behaviour after the guidance, which is crucial in retaining environmental benefits from behavioural changes.

## 3. Additional Workload Caused by Eco-Driving and Drivers’ Motivation

Existing studies conducted surveys to quantify the additional workload added by eco-driving instructions to investigate the cognitive load and acceptance of different types of eco-driving assistance. The subjective mental workload can be scored by the NASA Task Load Index (TLX) [83], and system acceptance can be measured by the System Acceptance Scale (SAS) of Van der Laan, which uses Usefulness and Satisfaction as metrics [83]. For example, Heyes et al. [77] adopted the SAS to evaluate drivers’ acceptance of the real-time driving advice provided by an in-vehicle system. The scale of Satisfaction significantly improved after using the system, while the scale of Usefulness did not. The positive attitude towards eco-driving assistance also led to less fuel consumption (4.01%) than those who did not support the system. Hibberd et al. [63] compared the workload among different types of dynamic sensory notifications. The results of SAS and NASA-TLX indicated that the haptic system is less distractive, with higher levels of Usefulness and Satisfaction than a visual–auditory assistance device.

In addition to survey-based experiments, real-time physiological measurement has been used in previous research to quantify the additional physiological workload caused by eco-driving. For instance, Ruscio et al. [84] measured heart rate, blood volume pulse, and high-frequency power of heart rate to quantitatively present the workload change when providing in-vehicle eco-driving assistance to drivers who had not used it before. The measurements showed higher cognitive loads when drivers were instructed to follow eco-driving assistance, introducing potential driving risks. Ahlstrom and Kircher [65] analysed glance behaviour with or without in-vehicle eco-driving guidance. The number and duration of glances varied when driving on different types of road, while mirror glances were reduced due to the in-vehicle guidance introducing more mental workload and higher risks during driving.

Limited studies investigated the inner motivation of drivers to implement improved driving behaviour. Existing research presents a significant variation in the effect of eco-driving guidance among drivers from the perspectives of both system acceptance and real-driving practice [85]. The effect of socio-demographic factors, such as age, gender, and education level, varies across studies, and the results can be different [45,86,87]. Lauper et al. [88] described the adoption of eco-driving as the consequence of two psychological processes: the formation of the behavioural intention and the process of putting the intention into practice. They found that socio-demographic characteristics only show minor effects on the intention of eco-driving. Instead, drivers’ attitudes towards eco-driving and perceived behavioural control were shown to be the strongest predictor of eco-driving intention, and the action control was the strongest predictor of eco-driving practice. This study emphasizes that instead of socio-demographic factors, the inner motivation of drivers can be the most influential personal characteristic of eco-driving behaviour.

Environmental concerns, a positive attitude toward eco-driving, and an open mind to new technologies are possible inner motivation factors for drivers to practice on-road eco-driving [43,89]. However, the opposite results have also been found in existing studies. For example, McIlroy and Stanton [87] analysed the relationship between the attitude towards eco-driving and the actual practice of eco-driving through an online survey. The result showed that the knowledge of eco-driving and awareness of environmental issues do not necessarily cause eco-driving behaviour in the real world across genders, ages, and levels of education. A similar result was also revealed by Scott and Lawson [90], that drivers usually do not apply fuel-saving driving operations although they have related guidelines in mind, and a gap exists between eco-driving knowledge and practice. As was illustrated by Pampel et al. [91], driving interventions are required to maintain the intention to utilize eco-driving guidelines and put eco-driving into practice; otherwise, the drivers would not practice eco-driving behaviour in the real world. The studies mentioned above emphasize the challenge of encouraging eco-driving practices, which may need specific designs of eco-driving guidance systems and the possible involvement of intrinsic and extrinsic motivations with the consideration of the psychological processes of human beings.

## 4. Discussion

### 4.1. Comparison with Previous Eco-Driving Reviews

Existing studies have reviewed current eco-driving research from multiple perspectives. Sivak and Schoettle [92] systematically reviewed drivers’ decisions ranging from vehicle choice, maintenance, route selection, and operational driving behaviour, and the study compared their effect on energy consumption; Alam and McNabola [34] comprehensively investigated the claimed benefits of existing eco-driving policies and technologies and summarized the limitations of them.

In terms of technical methodologies, Zhou et al. [93] summarized fuel consumption models suitable for eco-driving; Singh and Kathuria [94] reviewed driving behaviour profiling methods and their applications in eco-driving feedback. Mintsis et al. [94] reviewed existing research on eco-driving control optimization methods. Although some of the articles reviewed in Mintsis et al. implemented eco-driving advice to human drivers on real roads, this review emphasized the application of optimized control at signalized intersections.

Regarding review articles focusing on human-driver eco-driving guidance, Allison and Stanton [35] summarized a wide range of eco-driving guidance systems, including eco-driving training and feedback. Their benefits and problems were qualitatively described. In addition, this work and similar review work such as that of Huang et al. [95] focused on the design of a guidance system, with mostly highlighted the fuel-saving effect of the design.

The research review of this paper distinguished itself from previous review work by comparing the effect of different designs of eco-driving guidance, with a focus on factors that influence the system’s effectiveness and acceptance. The effect of drivers’ characteristics, including their acceptance, motivation to practice eco-driving, and driving workload, was also reviewed. In addition, this work reviewed the effect of emerging technologies on drivers’ attitudes towards eco-driving, such as integration with V2X communication and gamification elements.

### 4.2. Challenges in Eco-Driving Guidance

#### 4.2.1. Difficulties in Transferring Eco-Driving Knowledge to Eco-Driving Practice

Eco-driving is not a natural driving style and has not become a general target in current driving training courses. Therefore, even if drivers may have eco-driving knowledge in mind [91] or have received text messages about their eco-driving performance [96], they would not implement eco-driving unless they are asked to. Current eco-driving guidance mostly plays the role of “reminding” drivers by warning them of their inappropriate behaviour or providing basic suggestions, while the question of how to provide practical and instructive information is still under investigation. A common agreement on the influencing factors of eco-driving guidance has not been reached. In addition, a great number of questions remain to be answered: for example, how socio-demographic factors influence the guidance result, which type of guidance is more suitable for a specific group of drivers, and how to generate adaptive driving suggestions according to instantaneous traffic conditions and personal driving habits. The long-term effect of eco-driving guidance remains debatable: most studies present a fading impact of either static or dynamic eco-driving guidance, leading to an inconsistent energy-saving improvement (for example, [28,35,45,55,82]).

Another essential knowledge gap is the content of eco-driving guidance that can maximally lead to behavioural change in real-world practice. Drivers prefer simple, clear, and informative eco-driving suggestions [64,97,98,99]. Currently, limited articles have investigated drivers’ perceived acceptance of different guidance information. The contents of driving suggestions have seldom been compared in real-world driving experiments, especially with the background of semi-automation and telecommunication technologies.

Drivers’ trust in the guidance information, which affects their acceptance [100], should be improved through more precise and practical driving suggestions instead of general guidelines such as the Golden Rules. In this sense, vehicle sensing devices, such as cameras and the LiDar, need to be incorporated. It is necessary to retrieve information on road traffic conditions and the movement of surrounding vehicles in order to generate optimal driving suggestions given the traffic condition. While different from CAV-incorporated eco-driving optimization, eco-driving suggestions provided to human drivers need to adjust the information in a timely manner in response to drivers’ feedback (i.e., their driving behaviour after receiving the guidance). The displayed eco-driving guidance should be evolved based on the optimal trajectory, drivers’ historical habits, and feedback, so that the learning path can be shortened.

#### 4.2.2. Improving Experimental Design That Balances Safety and Effectiveness

Although recent studies have proposed customized learning paths based on drivers’ current driving behaviour to improve the acceptance level [54], the variation of tested traffic conditions, the characteristics of participating drivers, and the adaptive driving guidance algorithms still need to be investigated. In addition, a comprehensive experiment on different types of optimization algorithms is advised to maximize the effectiveness of the eco-driving guidance. On top of that, we suggest a standardized experiment design method so that the results of different studies can be comparable.

Considering safety issues, current research utilizes driving simulators for experiments (for example, [53,63,64,80,81,101]). However, due to short driving distances, testing durations, and limited traffic scenarios, the driving simulator may not be able to record enough influencing factors that reflect both inner motivation and learning progress with regard to eco-driving behaviour. In addition, since drivers may modify their aggressive driving behaviour when recognizing the existence of a testing device, results from a driving simulator can be much better than that of on-road driving in terms of energy savings and emission reductions. On-road tests are still necessary to develop an adaptive and easy-to-follow eco-driving assistance system, while the safety issue caused by additional workloads from reading eco-driving guidance should be considered. In future studies, driving simulator experiments can be used as a prior experience, followed by in-field studies, to capture more complicated traffic conditions and corresponding drivers’ reactions. Future eco-driving guidance should also balance environmental benefits and driving safety to avoid potential accidents due to additional workloads.

#### 4.2.3. Encouraging Drivers’ Acceptance through Gamified Designs

The popularity of gamification introduces new presence formats of eco-driving guidance. Gamified elements commonly involved in existing designs are points and leaderboards, or in other words, scores or rankings of their eco-driving behaviour among all participants. However, due to the complexity of traffic conditions and the diversity of vehicle specifications, energy consumption levels can be greatly varied among drivers who have similar driving habits, and the ranking-based scoring algorithm may be unfair. This further leads to the adaptation of scoring algorithms and the acceptance of gamified eco-driving guidance.

Previous research has tried to use supervised learning to differentiate traffic conditions based on the geographic information and temporal factors of road segments [102], while the adaptiveness and scalability of the classification algorithm need to be improved. Unsupervised learning algorithms implemented in risky behaviour detection, such as that proposed by Castignani et al. [103], can be introduced to classify drivers’ behaviour and identify traffic conditions around a targeted vehicle. In this case, a fair driving score specific for a particular group of drivers on certain driving conditions can be generated, which may adaptively encourage behavioural changes towards eco-driving.

## 5. Conclusions

This paper categorizes existing research about eco-driving guidance into two major types, static training and dynamic assistance, based on the use of real-time driving data to generate driving suggestions. Representative studies of both types are presented, and their effects on energy consumption and emissions are compared.

By summarizing the results of eco-driving experiments and surveys, challenges are proposed in this study. We conclude that static eco-driving training cannot ensure a sustainable change in driving behaviour, while whether regular incentives help maintain the training effect or not is debatable across studies. As “semi-dynamic” guidance, a periodic driving report leads to energy savings and emission reductions in a longer time span than pure training. The consequent behavioural change is affected by the feedback’s frequency and content. Comparing different sensory devices in dynamic guidance, it is shown that haptic touch feedback is less distractive with higher users’ acceptance than auditory and visualization devices. In terms of drivers’ characteristics, inexperienced and unprofessional drivers are more likely to adopt driving suggestions and change their behaviour, although exceptions exist (see [104]). Socio-demographic factors are associated with the adaptiveness of the eco-driving guidance, while the influence is varied, and the intentions are unclear. Most existing studies suggest that environmental concerns and a positive attitude towards eco-driving improve the acceptance level of eco-driving guidance, while drivers still need reminders to put their eco-driving knowledge into practice. A systematic design of eco-driving guidance, including the suggestions’ content, illustration, and human–machine interaction, should be examined to assess the motivation factors of drivers’ intention to accept and practice these suggestions. In addition, to improve the effectiveness, personalized recommendations based on historical driving data and the learning curve should be investigated.

## Figures and Tables

**Table 1:** Effects of static eco-driving guidance on energy savings and emission reductions *.

Study	Vehicle Type **	Guidance Type	Guidance Design	Add-On Options	Experiment Type	Effect Sustaining	Energy-Saving/Emission Reduction Effects
[21]	Medium-class vehicles	Static	Courses	/	On-road test	Immediate	On average, CO_2_ reduction by 1.7 kg per vehicle per day
[22]	Light-duty vehicles	Static	Courses	/	On-road test	Immediate	On average, 12% fuel saving
[23]	Private vehicles	Static	Courses	/	On-road test, survey	1 week after the training	On average, the fuel economy was reduced by 0.894 km/L to 1.378 km/L
[24]	Resort vehicles	Static	Courses	/	On-road test	Five months	8% fuel reduction, 8% CO_2_ reduction
[19]	Private vehicles	Static	Courses, coaches	/	On-road test	Immediate	More than 10%
[25]	Buses	Static	Courses	/	Simulator, survey	Immediate, and 6 months after the training	11.6% after the training, 16.9% fuel savings after 6 months
[26]	Logistics trucks	Static	Courses	Monetary and non-monetary incentives	On-road test	Immediate, and 12 months after the training	Significant effects only when adding non-monetary incentives, while the effect fades afterwards
[27]	Heavy- and medium-duty trucks	Static	Courses	/	On-road test		A fuel reduction of 6.8% (in L/ton-100 km)
[28]	Light-duty vehicles	Static	Courses	/	On-road test	10 months after the training	Fuel savings of 4.6% on city roads and 2.9% on highway roads
[20]	Light-duty vehicles	Static	Courses	/	On-road test	12 weeks after the training	4.6% fuel savings per 100 km
[29]	Private vehicles	Static	Courses	/	On-road test	Immediate	On average, 6.3% fuel savings (CO_2_ reduction)
[30]	Light-duty vehicles	Static	Courses	/	Simulator	Immediate	8.3% CO_2_ reduction, 8.4% fuel savings
[31]	Light-duty vehicles	Static	Courses, interactive guide	/	Simulator	Immediate	Up to 12.38% CO_2_ reduction
[32]	Waste collection trucks	Static	Courses, coaches	/	On-road test	3 months before and after the training	Up to USD 18,507.55 per month of savings in fuel cost, 7.1% reduction in CO_2_-e emissions and local air pollutants
[33]	Post vans	Static	Courses	/	On-road test, survey	1 to 2 weeks after the training	Insignificant differences

Note: * This table only includes studies that measure energy savings and emission reductions. ** Unless noted, vehicles used in the experiments are fossil-fuel-powered.

**Table 2:** Effects of dynamic eco-driving guidance on energy savings and emission reductions *.

Study	Vehicle Type **	Guidance Type	Guidance Design	Add-On Options	Experiment Type	Experiment Duration	Energy-Saving/Emission Reduction Effects
[36]	Buses	Dynamic	Feedbacks	/	On-road test, survey	1.5 years	1.4–4.6% fuel savings
[37]	/	Dynamic	Feedbacks	/	On-road test	Depends on the feedback frequency	Sporadic feedback leads to more CO_2_ reduction than daily feedback
[38]	Buses	Dynamic	Visualized, coaches	/	On-road test, survey	6 weeks	6.8% fuel savings
[39]	Light commercial vehicles	Dynamic	Visualized, auditory	/	On-road test	2 weeks	On average, 7.6% fuel savings
[40]	Light-duty vehicles	Dynamic	Visualized, haptic	/	Simulator	Immediate	On average, 15.9% to 18.4% fuel savings
[41]	Light-duty vehicles	Dynamic	Feedback, visualized, auditory	/	Simulator	Immediate	5.37% CO_2_ reduction, 5.45% fuel savings
[42]	Electric light-duty vehicles	Dynamic	Visualized	/	On-road test	Immediate	8.9% energy savings
[43]	Light-duty vehicles	Dynamic	Feedback, visualized	/	On-road test	10 months	On average 3% to 6% CO_2_ reduction
[44]	Buses	Static and dynamic	Courses and visualized advice	/	On-road test	One year	7% fuel savings
[17]	Light-duty vehicles	Dynamic	Visualized	/	On-road test	Immediate	On average, 30% fuel savings
[45]	Light-duty vehicles	Dynamic	Feedback	/	On-road test	3 months	0.4% fuel savings, 9.3% CO_2_ reduction
[46]	Military vehicles	Dynamic	Feedback	/	On-road test	50 weeks	3–10% fuel savings
[47]	/		Feedback	Peer-ranking	On-road test	4 months	31% fuel savings of the analysed driver
[48]	Taxis	Dynamic	Feedback	Peer-ranking	On-road test	1 month	On average 4.5% fuel savings
[49]	Taxis	Dynamic and static	Courses, coaching, feedback	Peer-ranking	Simulator, on-road test	1 week	Up to 9.6% fuel savings
[50]	Trucks and light commercial vehicles	Dynamic and static	Courses, feedback, visualized	Peer-ranking	On-road test	2 months	5.5% fuel savings
[51]	Light-duty vehicles	Dynamic	Courses, visualized, auditory, haptic	/	Simulator, survey	Immediate	Up to around 22% fuel savings
[52]	Light-duty vehicles	Dynamic	Haptic	/	Simulator, survey	Immediate	11% fuel savings
[53]	Commercial vehicles	Dynamic and static	Courses, feedback	Monetary rewards, peer-ranking	Simulator	Immediate	Peer competition has a more significant effect on CO_2_ reduction
[54]	Buses	Dynamic	Visualized, auditory	/	On-road test	19 months in total	6.25% fuel savings
[55]	Trucks	Dynamic	Visualized, coaches	/	On-road test	3 months	4% fuel savings
[56]	Light-duty vehicles	Dynamic	Visualized	/	On-road test, simulator	Immediate	Up to 45%
[57]	Light-duty vehicles	Dynamic	Visualized	Monetary rewards, peer-ranking	Simulator	Immediate	4.7% fuel savings
[58]	Electric light-duty vehicles	Dynamic	Feedback	Monetary rewards, peer-ranking	On-road test, survey	2–3 months	On average, 1.02 to 2.99 kWh/100 km energy savings
[59]	Light commercial vehicles	Dynamic	Auditory	/	On-road test	Immediate	5–6% fuel savings, up to 65% emission reduction (Nitrogen Oxide)
[60]	Trucks	Static and dynamic	Courses, feedback	Non-monetary incentives	On-road test	One year in total	5.2% to 9% fuel savings

Note: * This table only includes studies that measure energy saving and emission reduction. ** Unless noted, vehicles used in the experiments are fossil-fuel-powered.

## Data Availability

Not applicable.
